# Individual and community-level determinants of neonatal mortality in Somalia: a multilevel analysis of the 2018–2019 demographic and health survey

**DOI:** 10.3389/fped.2025.1693062

**Published:** 2025-12-18

**Authors:** Awo Mohamed Kahie, Abdirahman Omer Ali, Nura Mohamed Omer, Suhaib Mohamed Kahie, Saralees Nadarajah, Nima Muhammad Omer

**Affiliations:** 1 School of Postgraduate Studies and Research, Amoud University, Borama, Somalia; 2 College of Health Sciences, School of Medicine and Surgery, Amoud University, Borama, Somalia; 3 Department of Mathematics, University of Manchester, Manchester, United Kingdom

**Keywords:** Somalia, mortality, neonatal, multilevel analysis, health

## Abstract

**Background:**

Somalia faces one of the world's highest neonatal mortality (NM) rates, representing a severe public health crisis. This study aimed to identify the individual and community-level determinants of NM and describe its geographic distribution in this fragile setting, using data from the 2018–2019 Somali Demographic and Health Survey (SDHS).

**Methods:**

This cross-sectional study analyzed a weighted sample of 7,519 live births from the 2018–2019 SDHS. A two-level multilevel logistic regression model was used to identify individual and community-level determinants of neonatal mortality and to assess regional variations.

**Results:**

The neonatal mortality rate (NMR) was 34.1 deaths per 1,000 live births. At the individual level, multiple births significantly increased the odds of death [Adjusted Odds Ratio (AOR) = 3.92; 95% CI: 2.35–6.56], while a preceding birth interval of ≥2 years was protective, reducing mortality odds by 50% (AOR = 0.50; 95% CI: 0.39–0.66). Female newborns had 26% lower odds of mortality than males (AOR = 0.74; 95% CI: 0.58–0.95). At the community level, home delivery was a major risk factor, increasing the odds of neonatal death by over 50% compared to facility-based delivery (AOR = 1.52; 95% CI: 1.04–2.22). The analysis also revealed significant geographic disparities, with some regions showing substantially lower mortality risk than others, notably Hiraan (AOR = 0.15) and Banadir (AOR = 0.23) compared to Awdal.

**Conclusion:**

Neonatal mortality in Somalia is alarmingly high and inequitably distributed, driven by a combination of biological risks, healthcare access failures, and regional disparities. Interventions must prioritize increasing access to and utilization of health facilities for childbirth, strengthening family planning services to promote healthy birth spacing, and enhancing specialized care for high-risk newborns, particularly in regions identified with the highest mortality burden. These targeted strategies are essential to reduce preventable newborn deaths in Somalia.

## Introduction

Neonatal mortality, the death of a live-born infant within the first 28 days of life, remains a critical public health challenge, with 2.4 million infants lost globally in 2020 alone (1–3). The burden of these deaths is disproportionately borne by low- and middle-income countries (LMICs). In 2018, approximately 99% of the 2.5 million neonatal deaths occurred in LMICs, with Sub-Saharan Africa and South Asia together accounting for 79% of this total (4, 5). Infants born in Sub-Saharan Africa face a tenfold higher risk of dying in their first month compared to those in high-income nations (5).

The neonatal mortality rate (NMR) in Sub-Saharan Africa was 27 deaths per 1,000 live births in 2022, a figure that starkly contrasts with the Sustainable Development Goal (SDG) target of reducing the NMR to below 12 by 2030 (6–9). Most of these deaths are concentrated in the first week of life, with primary causes including premature birth, complications during childbirth (e.g., asphyxia), infections, and congenital anomalies (10, 11). These preventable conditions persist as major threats; for instance, Ethiopia's 2016 DHS reported an NMR of 29, showing slow progress, while other countries like Congo and Pakistan reported rates as high as 47 and 49, respectively (3, 12).

Somalia, a nation marked by decades of conflict and a fragile health system, faces one of the world's most severe neonatal mortality crises, with rates estimated between 38 and 40 deaths per 1,000 live births (13, 14). This alarmingly high rate reflects deep-seated challenges in healthcare access and quality. Well-established risk factors from other low-income settings—such as multiple births, short birth intervals, low maternal education, and lack of skilled delivery care—are likely exacerbated in the Somali context, impacting the social, economic, and psychological well-being of families (15–18). Previous studies in the region have consistently linked neonatal mortality to a complex interplay of factors, including maternal health, delivery practices, and socioeconomic status (1, 19–21).

Despite the scale of the problem, there is a scarcity of comprehensive, nationally representative studies analyzing the drivers of neonatal mortality in Somalia. Much of the existing research is localized or facility-based, failing to disentangle individual-level risks from the broader community and geographic factors (12–14). This study addresses this critical gap by applying a multilevel analysis to the 2018–2019 Somali Demographic and Health Survey (SDHS). This approach is uniquely suited to the hierarchical nature of DHS data, allowing for a robust examination of both individual and community-level determinants. Our objective is to identify these key determinants and map the geographic distribution of neonatal mortality, thereby providing critical evidence to guide targeted and effective public health interventions in Somalia.

## Methods and data

### Study setting, design, and area

This study employed a population-based, cross-sectional design using secondary data from the 2018–2019 Somali Health and Demographic Survey (SHDS). The SHDS is the first comprehensive, nationally representative survey of its kind in Somalia in over three decades, providing data on the five years preceding its collection. The survey was designed to be representative of urban, rural, and nomadic populations nationwide. However, due to significant security constraints during the data collection period, the Lower Shabelle and Middle Juba regions were inaccessible and are consequently excluded from this analysis. The SHDS contains detailed birth histories and information on child survival, making it an invaluable resource for investigating neonatal mortality determinants in the Somali context.

### Data source and sampling

We utilized data from the 2018–2019 Somali Health and Demographic Survey (SHDS), the first comprehensive demographic and health survey conducted in the country in over three decades. The SHDS was implemented by the Directorate of National Statistics of the Federal Government of Somalia, with data collection occurring between late 2018 and early 2019. This survey is often referred to as the “2020 SHDS” due to its publication year.

The survey employed a two-stage stratified cluster sampling methodology to ensure a nationally representative sample. In the first stage, Enumeration Areas (EAs) were selected from sampling frames with probability proportional to size. In the second stage, a fixed number of households were systematically selected from each EA. The sampling frame was stratified to represent urban, rural, and nomadic populations. After the exclusion of insecure areas, a total of 47 sampling strata were established for the final survey. The SHDS provides robust, high-quality data on health, nutrition, and demographic indicators essential for policymaking and program monitoring in Somalia.

### Sample size

The initial dataset comprised an unweighted sample of 7,671 live births that occurred in the five years preceding the survey. After excluding 152 cases with missing data on key variables necessary for the multilevel analysis (e.g., place of delivery, maternal education, or region), and applying the individual sample weights to account for the complex survey design, the final weighted analytical sample for this study consisted of 7,519 live births. These data were sourced from 16 geographic regions across Somalia. This weighting process ensured national representativeness for all subsequent statistical analyses.

### Study variables

The primary outcome variable for this study was neonatal mortality, defined as the death of a live-born infant within the first 28 days of life, in line with World Health Organization (WHO) guidelines (2). This variable was derived from the date of birth and the age at death of the infant. For the analysis, it was coded as a binary variable, where “1” indicated a neonatal death and “0” indicated that the infant was alive at the end of the neonatal period.

Explanatory variables were selected based on a comprehensive literature review of established risk factors for neonatal mortality in low- and middle-income countries, particularly in Sub-Saharan Africa, and their availability within the SDHS dataset. These factors have consistently been identified in previous studies as significant individual and community-level determinants of child survival (1, 19, 21, 22). Following established frameworks for child survival, these factors were categorized into two main levels: individual-level and community-level factors.

Individual-level factors encompassed characteristics related to the mother, the child, and the household.

Maternal characteristics included age group (15–29, 30–39, and 40–49 years), educational attainment (No Education, Primary, Secondary and Higher), and employment status in the last 12 months (Yes/No). Maternal reproductive history was captured through the total number of births (0–5 or 6 or more) and the preceding birth interval (<2 years or ≥2 years).

Child-specific factors consisted of sex (Male/Female), birth type (Single/Multiple), birth order (1–2, 3, or 4+), and the mother's perception of the infant's size at birth (Large, Normal, or Small).

Household characteristics included the total number of members (≤4 or >4 members) and the household's economic status as determined by the wealth index (Poor, Middle, or Rich). The categorization of household size into “≤4” and “>4” members was chosen to reflect commonly used thresholds in demographic health surveys that distinguish between smaller, potentially more resource-rich households, and larger households where resource dilution and increased exposure to infectious diseases may become more pronounced. This categorization aligns with previous studies in similar contexts and aims to capture the potential impact of household density on child survival outcomes.

Community-level factors represented the broader geographic, environmental, and health service contexts. These were grouped into two sub-categories.

Geographic and environmental factors included the administrative region of residence (16 regions), the type of settlement (Urban, Rural, or Nomadic), and the household's primary source of drinking water (Improved or Unimproved). Improved water sources typically include piped water into the dwelling, yard, or plot, public taps/standpipes, tube wells/boreholes, protected dug wells, protected springs, and rainwater. Unimproved sources generally refer to unprotected dug wells, unprotected springs, carting with animals, tankers, and bottled water when the primary source for bottling is unimproved.

Health service access and utilization indicators included the mother's perception of distance to a health facility (“Big Problem” or “No Problem”), the place of delivery (Health Facility or Home/Other), and the type of assistance during childbirth, classified as either Skilled (doctor, nurse, midwife) or Unskilled (traditional birth attendant, relative, or other).

### Data analysis

All data processing and statistical analyses were conducted using Stata statistical software version 16 (StataCorp, 2019). To account for the two-stage stratified cluster sampling design of the survey and to ensure the results were nationally representative, all analyses were weighted using the individual sample weight variable (v005) provided in the SHDS dataset. Descriptive statistics, including frequencies and percentages, were used to summarize the sociodemographic and reproductive characteristics of the participants, as well as the outcome variable, neonatal mortality.

Given the hierarchical nature of the SHDS data, where individuals (Level 1) are nested within communities or enumeration areas (Level 2), a two-level multilevel binary logistic regression model was employed. This approach correctly handles the non-independence of observations within clusters (i.e., intra-cluster correlation) and allows for the simultaneous examination of both individual and community-level predictors of neonatal mortality.

A four-stage model-building process was followed. First, a null model (Model 0) containing no predictor variables was fitted to assess the extent of community-level variance by calculating the Intraclass Correlation Coefficient (ICC). An ICC of 5% or greater justified the use of a multilevel approach. Next, Model I was fitted with only individual-level variables, followed by Model II, which included only community-level variables. Finally, Model III, the full model, was fitted containing both individual and community-level variables that were significant in the bivariate analysis. Variables with a *p*-value <0.25 in the bivariate analysis were considered candidates for inclusion in the multivariable models, a common practice to minimize the exclusion of potentially important variables that may show significance in a multivariable context (23, 24).

In the final model (Model III), Adjusted Odds Ratios (AORs) with 95% Confidence Intervals (CIs) were reported to measure the association between the predictor variables and neonatal mortality. A *p*-value <0.05 was used to declare statistical significance. Measures of variation (random effects) were assessed using the Median Odds Ratio (MOR) and Proportional Change in Variance (PCV). The final model's goodness-of-fit was assessed using Deviance and the Akaike Information Criterion (AIC), with the model having the lowest values being selected as the best-fitting model for the data (1, 25).

The ICC indicates the share of total variance due to cluster variation:

$$ICC=\frac{\sigma^{2}}{\sigma^{2}+\frac{\pi^{2}}{3}}$$

where *σ*^2^ represents cluster variance.

The MOR reflects the odds ratio between clusters with high and low neonatal mortality risk when randomly selecting two neonates from different clusters:

$$\mathrm{MOR}=\exp\left(\sqrt{2\cdot \sigma^{2}\;\cdot 0.6745\;}\;\right)\;\approx \exp(0.95\;.\;\delta )$$

PCV measures the variation in neonatal mortality explained by the final model (including individual and community factors) compared to the null model (without predictors):

$$\mathrm{PCV}=\frac{\mathrm{var}(\mathrm{null}\;\mathrm{model})-\;\mathrm{var}(\mathrm{full}\;\mathrm{model})}{\mathrm{var}(\mathrm{null}\;\mathrm{model})}$$

Models I, II, and III represent multilevel analyses with varying factors, and comparisons were made based on deviance.

The AOR with a 95% CI was reported to evaluate the association between neonatal mortality and independent variables. Pseudo linear regression assessed multicollinearity, yielding a mean Variance Inflation Factor (VIF) of less than 5 (21).

## Results

### Sample characteristics and descriptive analysis

The final weighted sample for this study comprised 7,519 live births. The overall neonatal mortality rate (NMR) was 34.1 deaths per 1,000 live births (95% Confidence Interval: 30.2–38.4). This high mortality rate occurred within a population characterized by a convergence of profound individual and community-level risk factors, which are detailed in Table 1. As shown in Table 1, an overwhelming majority of mothers had no formal education (85.22%) and were from households in the poorest wealth quintile (46.45%). These factors were reflected in health-seeking behaviors, as most deliveries occurred at home (83.60%) and were overseen by unskilled birth attendants (72.64%). Structural barriers further compounded these risks, with two-thirds of women (66.06%) reporting that distance to a health facility was a major problem. The obstetric profile was also high-risk, characterized by high parity (38.93% of mothers had ≥6 births) and short birth intervals (56.97% of births occurred <2 years after the preceding one), contributing to a notable proportion of small-for-size newborns (10.07%).

**Table 1:** Sociodemographic and health characteristics of the study population (*n* = 7,519).

Variables	Categories	Weighted frequency (*n*)	Percentage (%)
Community-Level Factors			
Region	Awdal	102	1.36
	Woqooyi Galbeed	491	6.53
	Togdheer	266	3.54
	Sool	194	2.58
	Sanaag	272	3.62
	Bari	528	7.02
	Nugaal	263	3.50
	Mudug	544	7.24
	Galgaduud	511	6.79
	Hiraan	256	3.40
	Middle Shabelle	494	6.57
	Banadir	1,891	25.15
	Bay	567	7.54
	Bakool	257	3.42
	Gedo	387	5.15
	Lower Juba	496	6.59
Residence	Rural	1,931	25.68
	Urban	4,648	61.81
	Nomadic	940	12.50
Healthcare Access (Distance)	Big Problem	4,967	66.06
	No Problem	2,552	33.94
Water Access	Improved	4,873	64.81
	Unimproved	2,646	35.19
Place of Delivery	Health Facility	1,233	16.40
	Home and others	6,286	83.60
Birth Attendance	Skilled	2,057	27.36
	Unskilled	5,462	72.64
Individual-Level Factors			
Maternal Age Group	15–29 Years	4,303	57.23
	30–39 Years	2,812	37.39
	40–49 Years	404	5.38
Maternal Education	No Education	6,408	85.22
	Primary	899	11.95
	Secondaryand Higher	212	2.82
Wealth Index	Poor	3,493	46.45
	Middle	1,464	19.47
	Rich	2,562	34.07
Household Size	≤4 members	2,505	33.32
	>4 members	5,014	66.68
Maternal Employment (last 12 months)	Yes	71	0.94
	No	7,448	99.06
Number of Births	0–5 Births	4,592	61.07
	6 or More Births	2,927	38.93
Child's Sex	Male	3,965	52.73
	Female	3,554	47.27
Birth Type	Single	7,358	97.86
	Multiple	161	2.14
Size at Birth	Large	699	9.30
	Normal	6,062	80.63
	Small	758	10.07
Birth Order	1–2	2,958	39.34
	3	1,178	15.67
	4+	3,383	45.00
Preceding Birth Interval	<2 Years	4,284	56.97
	≥2 Years	3,235	43.03

## Bivariate analysis of factors associated with neonatal mortality

Table 2 presents the results of the bivariate analysis, which was conducted to identify potential predictors for inclusion in the multivariable model (*p* ≤ 0.25). Several factors showed a significant association with neonatal mortality. A strong association was observed with the Region of residence (*p* < 0.001), indicating significant geographic disparities. Obstetric factors were also highly significant, including Birth Type (*p* < 0.001) and Preceding Birth Interval (*p* = 0.001). Among household characteristics, Household Size (*p* = 0.015) and access to an unimproved Water Source (*p* = 0.018) were also associated with higher mortality. Other variables, including Number of Births, Place of Delivery, Birth Order, Size at Birth, and Child's Sex, met the inclusion criteria for the multivariable model. Notably, maternal education, wealth index, and maternal age were not significantly associated with neonatal mortality at the bivariate level and were excluded from further analysis.

**Table 2:** Bivariate analysis of factors associated with neonatal mortality.

Variable	Categories	Alive, *n* (%)	Death, *n* (%)	*χ*^2^ (*p*-value)
Community-Level Factors				
Region	Awdal	94 (92.12)	8 (7.88)	**87.20 (*p* < 0.001)**
	Woqooyi Galbeed	480 (97.69)	11 (2.31)	
	Togdheer	250 (93.99)	16 (6.01)	
	Sool	184 (94.83)	10 (5.17)	
	Sanaag	264 (97.18)	8 (2.82)	
	Bari	510 (96.63)	18 (3.37)	
	Nugaal	253 (96.36)	10 (3.64)	
	Mudug	519 (95.47)	25 (4.53)	
	Galgaduud	500 (97.78)	11 (2.22)	
	Hiraan	253 (98.68)	3 (1.32)	
	Middle Shabelle	479 (96.92)	15 (3.08)	
	Banadir	1,861 (98.41)	30 (1.59)	
	Bay	524 (92.34)	43 (7.66)	
	Bakool	245 (95.45)	12 (4.55)	
	Gedo	379 (98.06)	8 (1.94)	
	Lower Juba	467 (94.25)	29 (5.75)	
Residence	Rural	1,867 (96.70)	64 (3.30)	1.43 (*p* = 0.592)
	Urban	4,494 (96.68)	154 (3.32)	
	Nomadic	902 (95.94)	38 (4.06)	
Healthcare Access (Distance)	Big Problem	4,805 (96.73)	162 (3.27)	0.88 (*p* = 0.505)
	No Problem	2,458 (96.32)	94 (3.68)	
Water Access	Improved	4,735 (97.17)	138 (2.83)	14.30 (*p* = 0.018)
	Unimproved	2,528 (95.53)	118 (4.47)	
Place of Delivery	Health Facility	1,203 (97.60)	30 (2.40)	4.61 (*p* = 0.077)
	Home and others	6,060 (96.40)	226 (3.60)	
Birth Attendance	Skilled	1,984 (96.42)	73 (3.58)	0.27 (*p* = 0.681)
	Unskilled	5,279 (96.66)	183 (3.34)	
Individual-Level Factors				
Maternal Age Group	15–29 Years	4,164 (96.76)	139 (3.24)	1.25 (*p* = 0.683)
	30–39 Years	2,708 (96.30)	104 (3.70)	
	40–49 Years	391 (96.90)	13 (3.10)	
Maternal Education	No Education	6,189 (96.58)	219 (3.42)	0.94 (*p* = 0.726)
	Primary	867 (96.43)	32 (3.57)	
	Secondary and Higher	207 (97.74)	5 (2.26)	
Wealth Index	Poor	3,389 (97.02)	104 (2.98)	5.29 (*p* = 0.338)
	Middle	1,401 (95.74)	63 (4.26)	
	Rich	2,472 (96.50)	90 (3.50)	
Household Size	≤4 members	2,441 (97.44)	64 (2.56)	8.36 (*p* = 0.015)
	>4 members	4,822 (96.17)	192 (3.83)	
Maternal Employment	Yes	69 (97.39)	2 (2.61)	0.14 (*p* = 0.691)
	No	7,192 (96.59)	256 (3.41)	
Number of Births	0–5 Births	4,452 (96.96)	140 (3.04)	4.89 (*p* = 0.054)
	6 or More Births	2,811 (96.02)	116 (3.98)	
Child's Sex	Male	3,817 (96.27)	148 (3.73)	2.71 (*p* = 0.211)
	Female	3,446 (96.95)	108 (3.05)	
Birth Type	Single	7,116 (96.71)	242 (3.29)	15.57 (*p* < 0.001)
	Multiple	147 (91.07)	14 (8.93)	
Size at Birth	Large	665 (95.08)	34 (4.92)	6.45 (*p* = 0.151)
	Normal	5,861 (96.67)	201 (3.33)	
	Small	738 (97.35)	20 (2.65)	
Birth Order	1–2	2,873 (97.13)	85 (2.87)	5.87 (*p* = 0.121)
	3	1,140 (96.81)	38 (3.19)	
	4+	3,250 (96.05)	133 (3.95)	
Preceding Birth Interval	<2 Years	4,107 (95.87)	177 (4.13)	16.15 (*p* = 0.001)
	≥2 Years	3,156 (97.55)	79 (2.45)	

The final multilevel logistic regression model (Model III in Table 3) identified significant predictors at both the individual and community levels.

**Table 3:** Results of multilevel logistic regression analysis for predictors of neonatal mortality.

Variable	Category	Model 0 (null model)	Model I (individual) AOR (95% CI)	Model II (community) AOR (95% CI)	Model III (full model) AOR (95% CI)
Individual-Level Factors					
Child Sex	Male		Ref		Ref
	Female		0.749 (0.587–0.955)*		0.743 (0.583–0.949)*
Birth Type	Single		Ref		Ref
	Multiple		4.020 (2.413–6.696)***		3.923 (2.346–6.561)***
Number of Births	0–5 Births		Ref		Ref
	6 or More Births		0.995 (0.677–1.461)		1.022 (0.693–1.507)
Birth Order	1–2		Ref		Ref
	3		1.185 (0.816–1.719)		1.154 (0.795–1.675)
	4+		1.410 (0.934–2.129)		1.345 (0.888–2.038)
Birth Interval	<2 Years		Ref		Ref
	≥2 Years		0.509 (0.391–0.665)***		0.503 (0.385–0.656)***
Household Size	≤4 Members		Ref		Ref
	4 Members		1.236 (0.955–1.600)		1.304 (1.005–1.690)*
Size at Birth	Large		Ref		Ref
	Normal		0.788 (0.526–1.179)		0.757 (0.504–1.137)
	Small		0.793 (0.469–1.339)		0.744 (0.439–1.261)
Community-Level Factors					
Region	Awdal			Ref	Ref
	Woqooyi Galbeed			0.553 (0.281–1.091)	0.577 (0.292–1.142)
	Togdheer			0.554 (0.272–1.128)	0.579 (0.283–1.182)
	Sool			0.635 (0.325–1.239)	0.622 (0.318–1.217)
	Sanaag			0.366 (0.183–0.732)**	0.354 (0.177–0.711)**
	Bari			0.529 (0.264–1.059)	0.534 (0.266–1.071)
	Nugaal			0.490 (0.244–0.982)*	0.520 (0.259–1.044)
	Mudug			0.575 (0.289–1.143)	0.599 (0.299–1.199)
	Galgaduud			0.312 (0.137–0.710)**	0.300 (0.131–0.686)**
	Hiraan			0.148 (0.052–0.419)***	0.147 (0.052–0.416)***
	Middle Shabelle			0.576 (0.283–1.174)	0.597 (0.291–1.223)
	Banadir			0.224 (0.104–0.481)***	0.225 (0.105–0.486)***
	Bay			0.937 (0.417–2.104)	0.921 (0.410–2.069)
	Bakool			0.436 (0.220–0.867)*	0.423 (0.212–0.841)*
	Gedo			0.263 (0.119–0.578)***	0.259 (0.117–0.572)***
	Lower Juba			0.512 (0.261–1.004)	0.503 (0.256–0.989)*
Place of Delivery	Health Facility			Ref	Ref
	Home and others			1.569 (1.076–2.287)*	1.522 (1.043–2.221)*
Water Access	Improved Water			Ref	Ref
	Unimproved Water			1.224 (0.937–1.600)	1.268 (0.967–1.661)

* *p* < 0.05.

** *p* < 0.01.

*** *p* < 0.001.

Model 0, the null model, contains no fixed effect predictors and its variance components are presented in Table 4.

At the individual level, biological and demographic factors were strong predictors. The odds of neonatal death were nearly four times higher for infants from multiple births compared to singletons (AOR = 3.92; 95% CI: 2.35–6.56). A preceding birth interval of two years or more was found to be highly protective, reducing the odds of death by 50% (AOR = 0.50; 95% CI: 0.39–0.66). Female newborns had 26% lower odds of mortality compared to males (AOR = 0.74; 95% CI: 0.58–0.95). In addition, larger household size (>4 members) was associated with 30% increased odds of neonatal death (AOR = 1.30; 95% CI: 1.00–1.69).

At the community level, the place of delivery was a critical determinant. Infants born at home or in other non-facility settings had over 50% higher odds of dying compared to those delivered in a health facility (AOR = 1.52; 95% CI: 1.04–2.22). Significant geographic disparities also persisted after controlling for other factors. Compared to the Awdal region (the reference category), several regions showed a significantly lower risk of neonatal mortality, including Hiraan (AOR = 0.15), Banadir (AOR = 0.23), Gedo (AOR = 0.26), Galgaduud (AOR = 0.30), Sanaag (AOR = 0.35), Bakool (AOR = 0.42), and Lower Juba (AOR = 0.50).

### Geographic disparities in neonatal mortality

**Figure 1:**
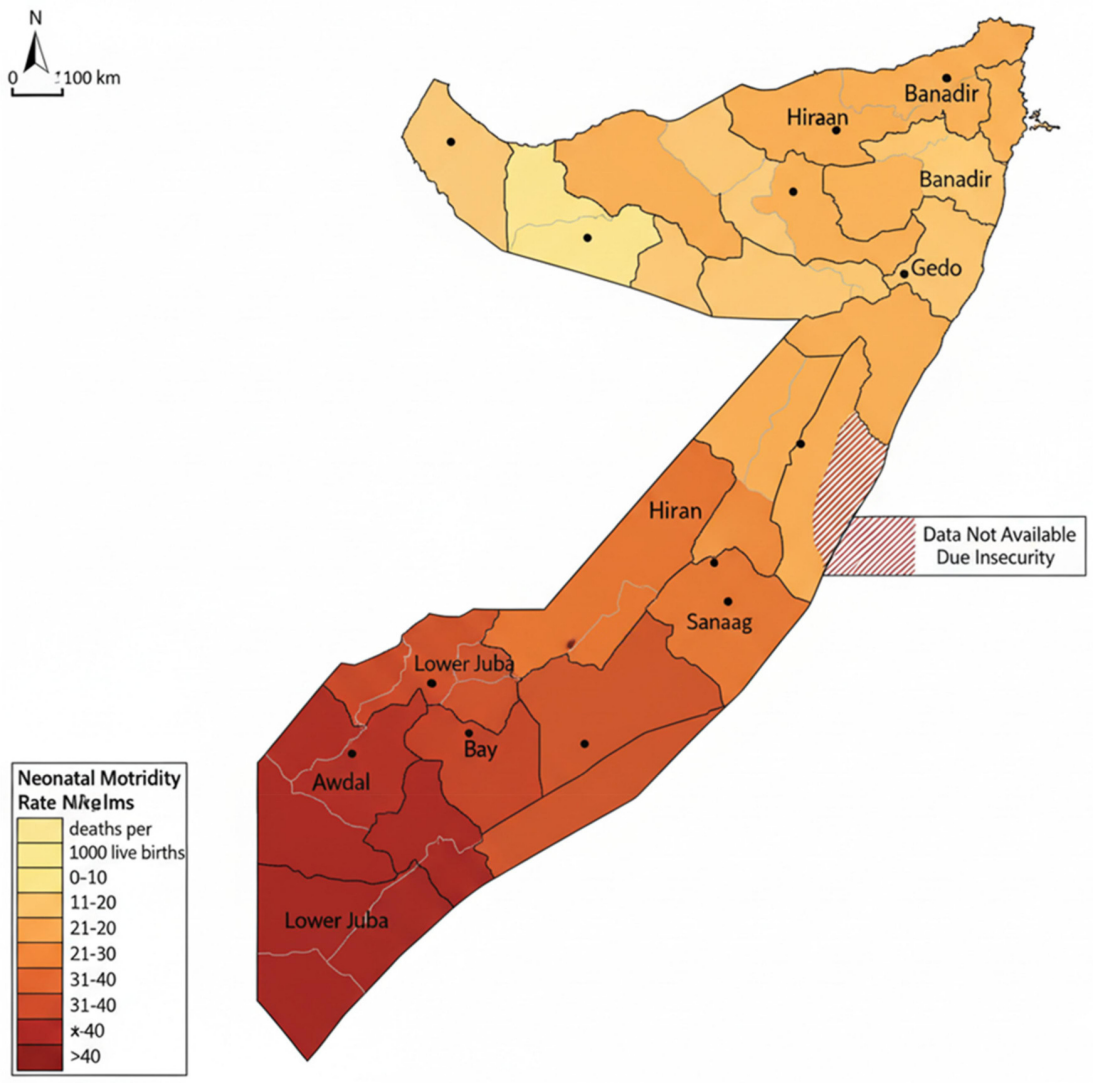
Geographic distribution neonatal mortality rate (NMR) across Somali regions, 2018–2019 SHDS.

The multilevel model revealed substantial geographic disparities in neonatal mortality across Somalia, which are visually represented in Figure 1. The map illustrates the spatial distribution of neonatal mortality risk, highlighting regions with a significantly higher or lower burden of death, even after accounting for individual and community-level factors.

## Random effect analysis and model comparison

The random effects analysis, summarized in Table 4, quantified the extent of clustering in neonatal mortality. The null model (Model 0) confirmed significant between-cluster variation (*τ*^2^ = 0.5946, *p* < 0.001). The Intraclass Correlation Coefficient (ICC) of 0.153 indicated that 15.3% of the total variance in neonatal mortality was attributable to cluster-level factors, justifying the use of a multilevel approach. The full model (Model III), incorporating both individual and community-level predictors, demonstrated the best fit, showing the greatest reduction in unexplained variance (*τ*^2^ = 0.3461) and the lowest Akaike Information Criterion (AIC) of 2,405.56. This confirms that a comprehensive model accounting for both levels of influence is essential for a robust understanding of neonatal mortality determinants in this context.

**Table 4:** Measurement of variation.

Measurement of variation	Null model (model 0)	Model I (individual factors)	Model II (community factors)	Model III (full model)
*τ* ^2^	0.5,946	0.5480	0.3913	0.3461
*P*-value	*P* < 0.001	*P* < 0.001	*P* = 0.0003	*P* = 0.0007
ICC	0.153	0.143	0.106	0.095
LR test	25.69	22.83	12.00	9.96
Model fit statistics				
AIC	2,472.73	2,421.40	2,459.03	2,405.56
BIC	2,486.62	2,483.91	2,590.99	2,586.13
−2Log-likelihood (Deviance)	2,468.73	2,403.40	2,421.03	2,353.56

## Discussion

This study provides the first comprehensive, nationally representative analysis of neonatal mortality in Somalia in over three decades, revealing an alarmingly high rate of 34.1 deaths per 1,000 live births. This rate is nearly three times the SDG target and underscores a profound public health crisis. Utilizing a multilevel approach, our findings demonstrate that this high mortality is driven by a complex web of individual biological risks, critical failures in healthcare access, and stark geographic disparities.

The finding that male neonates face a higher mortality risk aligns with a well-established biological disadvantage, which appears magnified in vulnerable populations (5, 26, 27). Similarly, the confirmation that multiple gestation is a critical determinant of neonatal mortality is consistent with established research (21, 22). These high-risk pregnancies are profoundly challenged by Somalia's fragile health system, where specialized care is scarce. Furthermore, the protective effect of longer birth intervals (≥2 years) reinforces the vital role of family planning in improving maternal and newborn survival outcomes, a conclusion widely supported by existing literature (28, 29).

A notable finding is the statistically significant association between larger household size and increased neonatal mortality, which is consistent with research from similar settings (19, 20, 30–33). This suggests that in high-density living environments, the pressures of resource dilution and heightened exposure to infectious pathogens may supersede the benefits of a larger social support network (34). In contrast, high maternal parity (≥6 births) did not emerge as a significant independent predictor. This suggests that in the Somali context, the timing and spacing of pregnancies—captured by the birth interval variable—may be a more critical determinant of neonatal survival than the cumulative number of births

The analysis did not find a statistically significant association between a mother's perception of birth size and neonatal death, which contradicts the well-documented risks of low birth weight (35, 36). This result should be interpreted with caution, as it is likely attributable to the subjective nature of the “maternal perception” variable, which is susceptible to recall bias and is not a direct measurement of birth weight. Therefore, our study cannot refute the established dangers of low birth weight, and further research using objective anthropometric data is needed (37–39).

At the community level, the finding that home births are associated with over 50% higher odds of mortality is a stark indicator of the life-saving importance of skilled care during delivery. This aligns with a vast body of evidence showing that access to emergency obstetric care in health facilities significantly reduces intra-partum related neonatal deaths (9, 40, 41). Furthermore, the profound impact of geographic location remained one of the most striking findings. Even after controlling for individual and household factors, the region of residence was a powerful, independent predictor of neonatal mortality. The significantly lower risk in regions like Hiraan and Banadir—the latter being the nation's capital with a higher concentration of health facilities—highlights the deep inequities in health resource distribution across the country.

## Strengths and limitations

The primary strength of this study is its use of a large, nationally representative dataset combined with advanced multilevel methods, providing robust insights for a data-scarce region. However, limitations must be acknowledged. The cross-sectional design prevents causal inference, and the data relies on maternal recall, which is subject to bias. Despite its age, the 2018–2019 dataset remains the most recent comprehensive national data available for Somalia. While the focus on Somalia may limit the direct applicability of these findings to other countries, the results offer critical insights for regional public health systems and neighboring nations facing similar challenges in fragile contexts. Finally, the exclusion of two insecure regions (Lower Shabelle and Middle Juba) means our findings may not be fully generalizable to the entire country, potentially underestimating the national mortality burden.

## Conclusion

This study finds that neonatal mortality in Somalia is alarmingly high and is shaped by a confluence of individual biological risks and community-level healthcare deficiencies, with profound geographic inequities defining the landscape of newborn survival. The findings identify home delivery, short birth intervals, multiple births, male sex, and larger household size as critical drivers of mortality, underscoring the urgent need for multi-pronged policy actions.

### Policy recommendations

Based on these findings, we offer several key recommendations. First, national health strategies must prioritize strengthening and promoting facility-based childbirth by mitigating financial, geographic, and cultural barriers to access. Second, maternal and newborn care must be enhanced for high-risk groups by integrating comprehensive family planning services to promote healthy birth spacing and strengthening the capacity of facilities to manage complications associated with multiple births. Finally, the significant regional variations in mortality necessitate geographically targeted interventions. The Ministry of Health and its partners must prioritize the allocation of resources and tailored public health initiatives to the regions identified with the highest mortality burden to address these inequities and reduce preventable newborn deaths.

## Data Availability

Publicly available datasets were analyzed in this study. This data can be found here: The SDHS data sets are publicly open and can be accessed using this link (https://microdata.nbs.gov.so/index.php/catalog/50). Further inquiries can be directed to the corresponding author.
